# Transformation strategies for stable expression of complex hetero‐multimeric proteins like secretory immunoglobulin A in plants

**DOI:** 10.1111/pbi.13098

**Published:** 2019-03-05

**Authors:** Jorge Palaci, Vikram Virdi, Ann Depicker

**Affiliations:** ^1^ Department of Plant Biotechnology and Bioinformatics Ghent University Gent Belgium; ^2^ VIB Center for Plant Systems Biology Gent Belgium; ^3^ Department of Biochemistry and Microbiology Ghent University Gent Belgium; ^4^ VIB Center for Medical Biotechnology Gent Belgium

**Keywords:** multimeric glycoprotein, secretory IgA, recombinant protein production, transgene expression variability, seed specific expression, molecular farming

## Abstract

Plant expression systems have proven to be exceptional in producing high‐value complex polymeric proteins such as secretory IgAs (SIgAs). However, polymeric protein production requires the expression of multiple genes, which can be transformed as single or multiple T‐DNA units to generate stable transgenic plant lines. Here, we evaluated four strategies to stably transform multiple genes and to obtain high expression of all components. Using the in‐seed expression of a simplified secretory IgA (sSIgA) as a reference molecule, we conclude that it is better to spread the genes over two T‐DNAs than to contain them in a single T‐DNA, because of the presence of homologous recombination events and gene silencing. These T‐DNAs can be cotransformed to obtain transgenic plants in one transformation step. However, if time permits, more transformants with high production levels of the polymeric protein can be obtained either by sequential transformation or by in‐parallel transformation followed by crossing of transformants independently selected for excellent expression of the genes in each T‐DNA.

## Introduction

Secretory immunoglobulin A (SIgA) is a heterodecameric Ig of 400 kDa that is predominantly involved in the protection of the mucosal surfaces against pathogenic infections. Application of pathogen‐specific SIgAs at the mucosal surfaces can provide immediate protection (Corthésy, 2010) but requires bulk amounts of the SIgA protein in the range of gram amounts per day and per patient. However, recombinant manufacturing of SIgA has been challenging because of its complex assembly. SIgA is composed of two monomeric IgAs (mIgAs) each made up of two heavy and two light chains, a joining (J‐)chain keeping the two mIgAs together in a dimeric IgA (dIgA), and the secretory component (SC), wrapped around the dIgA. Of the expression systems evaluated, plants have been shown to be the most successful platform for SIgAs (Ariaans *et al*., 2014; Ma *et al*., 1995). The bacterial and yeast systems currently do not efficiently secrete assembled Igs, and the levels of correctly post‐transcriptionally modified SIgAs are too low (Fischer *et al*., 2004; Vasilev *et al*., 2016). Also the few milligram production levels of SIgAs attained in mammalian or insect cell fermenters are not sufficient or cost effective to be translated into the clinic. In contrast, several different SIgAs have been expressed successfully in plants, and one of these SIgAs, that is, CaroRx, has been approved for use as medical device for dental care (Paul and Ma, 2011).

Plant‐made SIgAs can be produced either via transient expression or after stable genomic integration. The transient expression system has allowed successful extraction of SIgAs against rotavirus from leaves of *Nicotiana benthamiana* already five days post‐infiltration (Juarez *et al*., 2013) and its production capacity has been thoroughly validated, for instance, with the production of millions of vaccine doses in a month against H1N1 VLP influenza by the company Medicago (D'Aoust *et al*., 2010). However, for passive immunization applications, we believe that stable transformation is the most suited system for the cheap production of SIgA in plants because of the ease of scalability through the already established infrastructure of commercial varieties, the inheritable expression and the possibility to produce the recombinant protein in specific compartments and tissues, such as seeds, with advantageous properties (Stoger *et al*., 2014; Virdi *et al*., 2015). To obtain stably transformed SIgA‐producing plants, several strategies have been described. The first is the *Nicotiana tabacum* SIgA‐producing line obtained by sequentially crossing individual transformants with, the heavy chain, light chain, J‐chain and SC encoding genes (Ma *et al*., 1995). High SIgA levels were obtained, but three sequential crossings were needed to generate the stably transformed line containing the four different loci in homozygous condition. Using a single T‐DNA system in which all the four components required for SIgA production are clustered, significantly reduces the time to develop SIgA‐producing stable transformants as shown for Lemna (duckweed; Ariaans *et al*., 2014) and *Arabidopsis thaliana* (Arabidopsis; Nakanishi *et al*., 2017). However, the cloning process of this single T‐DNA can be challenging because of the large size of the construct. Previously, we evaluated a third strategy, which is based on the simultaneous cotransformation of all T‐DNAs with the required constructs for the in‐seed production of light‐chain devoid simplified SIgAs (sSIgAs) in Arabidopsis (Virdi *et al*., 2013). Four different variable domains (named V1, V2, V3 and V4) of llama heavy chain‐only antibodies (VHHs) against enterotoxigenic *Escherichia coli* (ETEC) were genetically fused to the Fc part of a porcine IgA. Upon triple cotransformation with *Agrobacterium* cultures bearing T‐DNAs with one of the four VHH‐IgA fusions, the J‐chain and the SC, each driven by the seed‐specific β‐phaseolin promoter, transformants producing sSIgA in seeds were identified. This strategy was much faster than the sequential crossing method, but the frequency with which triple cotransformants were obtained was about 1% of that for obtaining the single T‐DNA transformants. Later, X‐ray crystallography revealed that V1 and V2 bind to the same epitope, whereas V3 binds to another conformational epitope (Moonens *et al*., 2015). Moreover, V3 and V4 most likely bind the same epitope because they differ by a single amino acid (Virdi *et al*., 2013).

In comparison with a monomeric protein, the production of a recombinant hetero‐multimeric protein requires the appropriate contribution of several transgenes and their simultaneous transformation into the expression system (organism). Which transformation strategy to follow for the production of complex hetero‐multimeric proteins such as sSIgAs depends on several parameters such as time, desired protein expression level, tissue specificity and/or number of available selection markers. At first sight, cloning all the different sSIgA components in a single T‐DNA seems to be the most straightforward strategy. However, as the cloning process can be challenging because of the large size of the construct (about 20 kb), cotransformation seems to be a good alternative. On the other hand, sequential crossing allows a step‐by‐step screening for transformants with the best expression for each T‐DNA, but can be labour intensive because of the need to screen for double and triple homozygotes. Thus, when the aim is to produce complex hetero‐multimeric proteins in long‐life‐cycle plants, the proper choice of the transformation strategy plays a pivotal role in determining the feasibility of the project.

**Figure 1:**
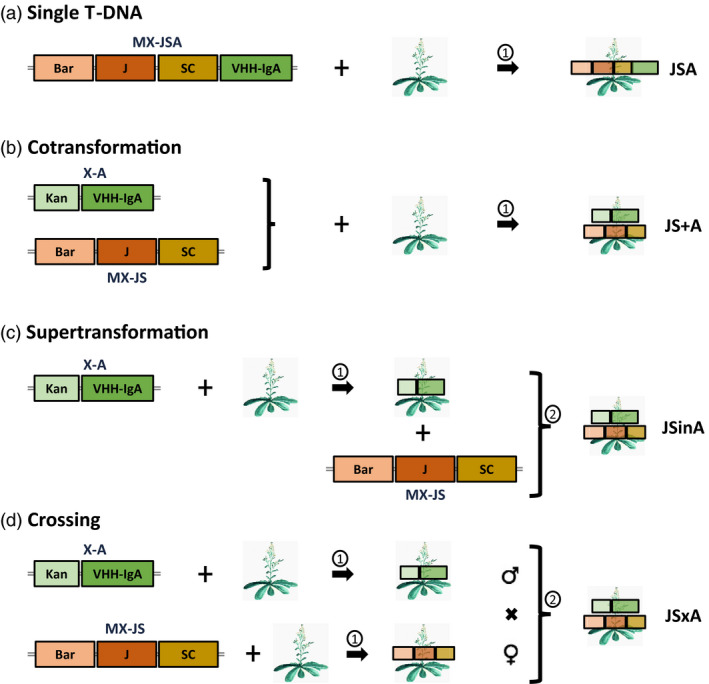
Different strategies evaluated for sSIgA production in *Arabidopsis thaliana*. In a first round of transformation (①), T‐DNAs MX‐JSA, MX‐JS and/or X‐A were introduced in wild‐type Col‐0 Arabidopsis plants via *Arabidopsis tumefaciens*‐mediated transformation. In the single T‐DNA (a) and cotransformation (b) strategies, no further transformation was required. In the supertransformation strategy (c), a second round (②) of plant manipulation was used to introduce the MX‐JS plasmid in a plant producing VHH‐IgA. In the parallel transformation and crossing strategy (d), a JS‐transformed line and a VHH‐IgA‐transformed lined were crossed. Wild‐type Arabidopsis plants are depicted with the image of an Arabidopsis plant, while transformed transformants are indicated by representing the T‐DNA on top of the plant. Bar, phosphinothricin herbicide resistance gene; Kan, kanamycin resistance gene; J, joining chain; SC, secretory component; MX, multiple gene expression vector; X, gene expression vector.

Here, we compared these three above‐mentioned strategies with a sequential transformation strategy (Figure 1), and evaluated the straightforwardness to obtain plants with high sSIgA accumulation levels. We used the V2 and V3 VHH‐IgA variants against ETEC developed by us previously (Virdi *et al*. 2013) and Arabidopsis as the perfect model to test the stable production of complex hetero‐multimeric proteins in plants because of its short life cycle. As such, we wanted to pinpoint the advantages and drawbacks for each of the strategies, and which transformation method to choose for the production of a SIgA and alike hetero‐multimeric protein in plants.

## Results

### Cloning of the sSIgA‐encoding components

To evaluate these alternative transformation strategies for sSIgA production in stably transformed Arabidopsis plants, different T‐DNA constructs were assembled, containing the three transgenes (i.e. VHH‐IgA, J‐chain and SC) required for sSIgA production in one T‐DNA or in two different T‐DNAs (Figure S1). Previously, we have designed four different VHH‐IgA antibodies against ETEC and expressed them in *A. thaliana* seeds to enable oral passive immunization of weaned piglets by adding them to the feed (Virdi *et al*., 2013). Here, we included two of them, V2 and V3, in the cloning and subsequent plant transformations to evaluate the reproducibility of our results. The J‐chain, SC and VHH‐IgA encoding sequences were each fused with the N‐terminal 2S2 signal peptide and the C‐terminal KDEL motif sequences for ER targeting and retention, respectively. The coding sequences were transcriptionally controlled by the 5′ β phaseolin (1475‐bp) promoter and the 3′ arceline (600‐bp) terminator to obtain seed‐specific expression (Morandini *et al*., 2011) like in our previous study, so we could use the best sSIgA producer for the V2 and V3 variant obtained after triple cotransformation by Virdi *et al*. (2013) as reference seed stocks.

For the single T‐DNA transformation strategy (Figure 1a), these three sSIgA expression cassettes were cloned in tandem (same transcriptional orientation) in a single T‐DNA construct (MX‐JSA, Figure S1a). By restriction characterization of individual *E. coli* clones, we found that some of them integrated a double copy of the VHH‐IgA cassette, and because this might result in a positive dosage effect, we included this “single T‐DNA double VHH‐IgA” (MX‐JSAA, Figure S1) construct in the comparative transformation efficiency analysis. Restriction analysis of the clones also demonstrated the frequent occurrence of a particular variant with missing fragments in the expected restriction pattern (Figure S2), and upon inspection, this corresponded to the loss of the J‐chain expression cassette in the pMX‐JSA and pMX‐JSAA T‐DNA vector plasmids (Figure S1a,b). These frequently observed deletions occurred not only upon transformation and establishment of the clones in *E. coli*, but also upon propagation of the *E. coli* and *Agrobacterium* clones.

For the other three transformation approaches (Figure 1b–d), the constant elements, being the J‐chain and SC, were clustered in one T‐DNA (MX‐JS; Figure S1c), and the variable VHH‐IgA, either V2 or V3, was cloned in the other T‐DNA (X‐A; Figure S1d).

Once the different T‐DNA constructs were obtained and transferred into *Arabidopsis tumefaciens*, all transformations were performed by floral dip in wild‐type Col‐0 plants as schematically depicted in Figure 1. T1 transformants were selected on phosphinothricin and/or kanamycin and grown to obtain T2 seed stocks, which were then screened for their sSIgA production level. For the crossing strategy, F2 hybrid seeds were analysed. For the heritability study, homozygous T3 seeds were analysed.

### Efficiencies of different transformation strategies to obtain sSIgA‐producing plants

If speed is an issue, the only two methods that need a single transformation step are gene stacking of the components in a single JSA or JSAA T‐DNA and cotransformation of the JS and VHH‐IgA T‐DNAs, referred to as JS+A (Figure 1a,b). The efficiency by which all three transgenes were integrated in a single transformant, independently of the VHH variant, was recorded for the two strategies. We obtained four times more transformants with the single JSA T‐DNA [2.38% (±1.47) of the T1 seeds] as compared with double selected JS+A cotransformants [0.52% (±0.36) of the T1 seeds].

In the supertransformation strategy (Figure 1c), two subsequent transformations are required to obtain the sSIgA‐producing plants: first single‐locus homozygous plants producing either VHH‐IgA or the constant components (J‐chain together with the SC) need to be selected, after which they are supertransformed with the other T‐DNA. Here, we supertransformed the already available lines with high levels of either the V2 or V3 VHH‐IgA variant (Virdi *et al*., 2013) with a JS T‐DNA containing the J‐chain and SC genes. In this way, 21 supertransformants were obtained and referred to as JSinA. The efficiency by which the new T‐DNA got integrated in the recombinant plants was 4.38% (±1.87).

For the crossing strategy (Figure 1d), 24 transformants with the JS T‐DNA, obtained with a frequency of 1.58% (±0.29) in the T1 generation, were selected and expression of the J‐chain and SC was screened by quantification in western blots (Figure S3). Highly variable levels of J‐chain and SC expression were observed between the JS transformants, and counter‐intuitively, also the relative levels of J‐chain and SC within each transformant were variable (Figure S3a). However, the J‐chain western blot assay revealed the predicted single band at about 20 kDa, the SC western blot showed a main band of 75 kDa corresponding to the glycosylated version (as shown later in the characterization of sSIgA) and a band at the expected molecular weight of 65 kDa, corresponding to the non‐glycosylated SC. Six transformants showing predominantly glycosylated SC in their western blot profile and relatively high expression of both SC and J‐chain were propagated to the T3 generation, and of those, four homozygous, single‐locus JS transformants were retained (Figure S3b). These were then crossed with the same VHH‐IgA‐producing lines as used in the supertransformation strategy to obtain sSIgA‐producing hybrid seeds referred to as JSxA. All the F1 hybrids obtained were resistant to both kanamycin and phosphinothricin, indicating the successful crossing of the lines.

To summarize (Table 1), 15–23 transformants were obtained by the single T‐DNA (JSA or JSAA) or the cotransformation (JS+A) strategies for both the V2 and V3 variants. In the sequential transformation strategy, 21 JS supertransformants of the VHH‐IgA expressing lines V2 and V3 were selected (JSinA), and for the crossing strategy, four hybrid F2 progeny seed stocks (JSxA) were obtained by crossing the V2 or V3 VHH‐IgA‐expressing line with the four best selected events from 24 JS transformants.

**Table 1:** Number (No.) of T2 transformants or F2 hybrids obtained for protein expression analysis and proportion (%) of them unable to assemble sSIgA despite of showing good VHH‐IgA accumulation levels, per transformation group and type of VHH‐IgA (V2 or V3)

Transformation group	V2		V3	
	No.	%	No.	%
JSA	21	14%	21	29%
JSAA	23	43%	21	29%
JS+A	15	0%	21	0%
JSinA	21	0%	21	0%
JSxA	4	0%	4	0%

### sSIgA levels in seeds obtained by different transformation strategies

The relative amounts of sSIgA recombinant protein production were determined by an sSIgA‐specific ELISA (Figure S4a), in which only the antigen‐binding, fully assembled sSIgAs are measured by detecting the complex with SC‐specific antibodies, while the antigen‐binding VHH‐IgA levels present as monomeric, dimeric and secretory IgAs were determined by a VHH‐IgA‐specific ELISA (Figure S4b).

**Figure 2:**
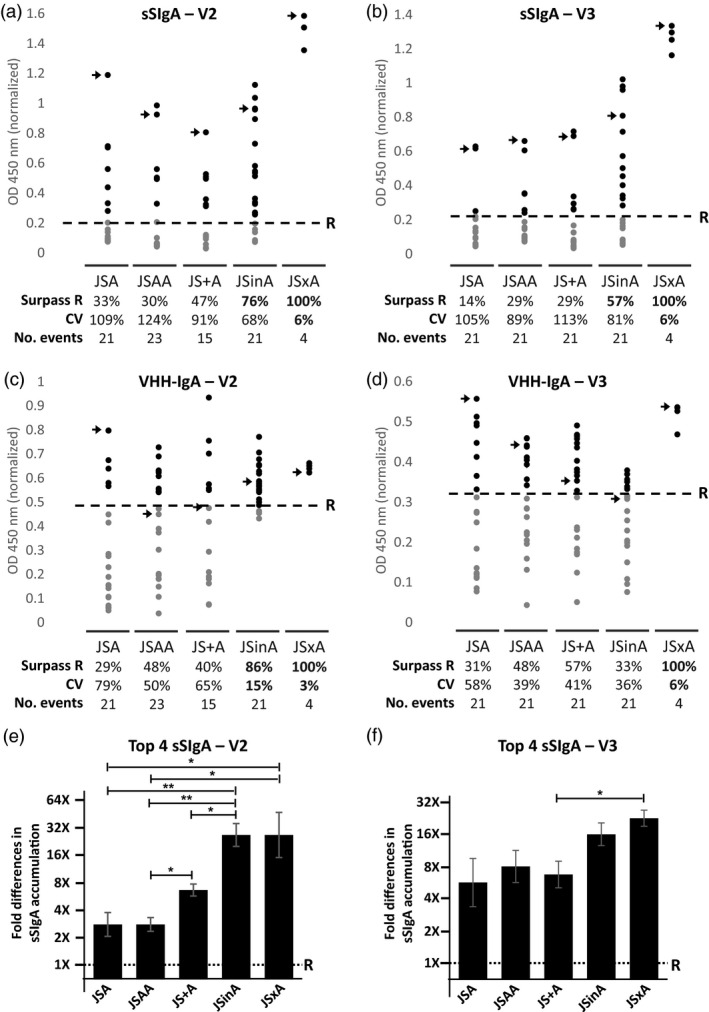
Relative ELISA‐based quantification of sSIgA and VHH‐IgA in seed extracts. Relative sSIgA content (a and b) and relative antigen‐binding VHH‐IgA content (c and d) in transformants obtained by different transformation strategies, for the V2 (left) and V3 (right) VHH‐IgA variants. The plotted OD values obtained for each transformant at a specific dilution in a sSIgA‐specific and a VHH‐IgA‐specific ELISA, respectively, were normalized using a reference (R) extract made from the best sSIgA‐producing lines obtained by Virdi *et al*. (2013) (sV2A8 for the V2 and sV3A40 for the V3 variant). The percentage of transformants that surpassed the reference sample (represented by a dashed line) in sSIgA or VHH‐IgA accumulation (Surpass R), the coefficient of variation (CV) and the number of transformants analysed (No. events) are indicated below each group. The samples that were further characterized by Western blot (Figure 3) are indicated with an arrow. (e and f) Comparison of the sSIgA levels from each transformation strategy by a titration experiment. Each bar represents the average relative sSIgA level of the four highest sSIgA‐accumulating transformants for each strategy as compared with the reference. The *Y*‐axis represents the dilution value by which the OD of a specific sample goes below the threshold, first calculated as a log2 value (±SEM) and then transformed into relative units compared with the dilution value recorded for the reference (1×). Error bars represent standard error of the mean. Statistical significance was evaluated by *T*‐test. *P < 0.05; **P < 0.01.

First, the levels of antigen‐binding sSIgA in seed extracts from transformants belonging to the different transformation groups were compared with those in reference seed stocks for the V2 and V3 variant, producing both VHH‐IgA antibodies to levels of 0.2% of seed weight and selected as the best sSIgA producers after triple cotransformation by Virdi *et al*. (2013). Because sSIgAs could not be purified from a mixture of monomeric, dimeric and secretory IgAs, it prevented us from performing absolute quantifications of the sSIgA content. Conversely, the relative quantification of the sSIgA content allowed us to determine the proportion of transformants in each transformation series that surpassed the sSIgA content of the reference seed stock. The proportion of V2 sSIgA transformants accumulating more sSIgAs than the reference was 33% for the JSA single T‐DNA, 30% for the JSAA single T‐DNA, 47% for the JS+A cotransformation, 76% for the JSinA supertransformation and 100% for the hybrids obtained by crossing (Figure 2a). The same trend was seen for the V3 sSIgA transformants: 14%, 29%, 29%, 57% and 100% for JSA, JSAA, JS+A, JSinA, and JSxA, respectively (Figure 2b). Thus, the supertransformation and crossing strategies provided clearly the largest proportion of highly producing sSIgA transformants. Among these, only the crossing strategy of selected events resulted in a low coefficient of variation (CV) for sSIgA production.

Second, the accumulation of antigen binding VHH‐IgA was determined in all V2 and V3 sSIgA transformants, independent of whether it was present in a monomeric, dimeric or assembled secretory format. For both the single T‐DNA and cotransformation approaches (JSA, JSAA and JS+A), a large variability in antigen‐binding VHH‐IgA accumulation levels could be observed (Figure 2c,d). The results show that in some groups of transformants, a positive correlation with the sSIgA and VHH‐IgA levels could be seen, like such as in the group of JSA(V2) transformants, but in other groups of transformants, there was no correlation with the levels of sSIgA and VHH‐IgA, such as in the JSAA(V3) group (Figure S5). This indicates that the levels of either of the three components can be limiting to obtain high sSIgA levels. Furthermore, and remarkably, a rather high percentage of up to 43% of the single T‐DNA transformants (JSA and JSAA) did not contain detectable amounts of assembled sSIgA (Table 1), whereas they did almost all contain antigen‐binding VHH‐IgA (Figure 2c,d). In contrast, all of the analysed seed extracts from JS+A or JSinA transformants expressing detectable levels of VHH‐IgA, produced also the assembled sSIgA in variable amounts. In comparison with the JSA and JS+A obtained seed stocks, the VHH‐IgA accumulation levels in supertransformed JSinA and JSxA crossed seed stocks were much more similar in the V2 variant, as pointed by their coefficient of variation (CV) (Figure 2c). A similar pattern was seen for the V3 VHH‐IgA levels, except for the JSinA transformants that experienced a moderate increase in the CV as compared with the V2 (Figure 2d).

Then, the four best sSIgA‐accumulating transformants from each strategy were chosen and the relative accumulation of sSIgA was compared in an ELISA titration experiment. We found that the V2 sSIgA accumulation levels ranged, on average, from more than double to almost 32‐fold higher than the reference line (Figure 2e). More specifically, the best JSA and JSAA transformants contained two‐ to fourfold more sSIgA accumulation than the reference line. For the JS+A cotransformants, the sSIgA levels were about eightfold higher, and among the JSinA supertransformants and JSxA hybrids almost 32‐fold higher sSIgA levels were found. Outliers from the supertransformation and crossing strategies even contained 64‐fold higher sSIgA levels than the reference line. Similar results were obtained for the four best V3 sSIgA expressors. In this case however, JSA and JSAA transformants reached slightly better accumulation levels (between four‐ to eight‐fold difference than the reference) and differences were not always statistically significant between these groups and the JSinA and JSxA transformants (Figure 2f).

### Molecular characterization of sSIgA‐expressing plants

**Figure 3:**
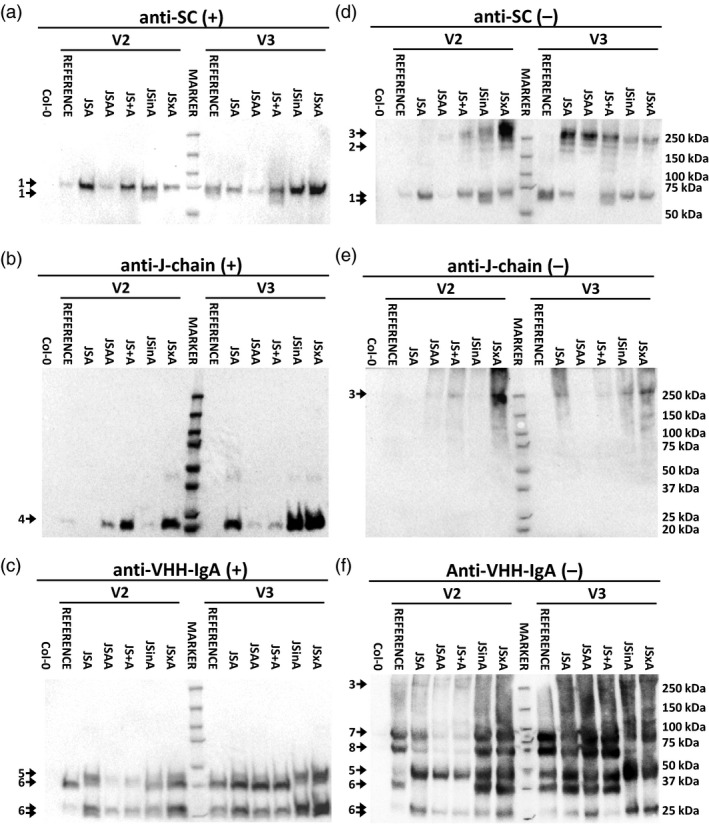
Western blot characterization of the selected transformants for each transformation strategy. Detection of SC (a and d), J‐chain (b and e) and VHH‐IgA (c and f) in a western blot after reducing (+) and non‐reducing (−) PAGE. The main bands, indicated with a number, most likely correspond with SC in different glycosylated forms (1), a postulated dimeric SC (2), the assembled sSIgA complex (3), J‐chain (4), a single polypeptide of VHH‐IgA (5) and truncated versions thereof (6), assembled simplified mIgA (7) and a truncated version of the mIgA (8).

The seed extracts of the best sSIgA transformants in each strategy were further analysed in different western blot setups. After sodium dodecyl sulfate‐polyacrylamide gel electrophoresis (SDS‐PAGE) of the seed extracts in reducing conditions, SC‐specific antibodies revealed a band at about 65 kDa corresponding to a non‐glycosylated form of SC and higher bands corresponding to glycoforms (Figure 3a) as shown by a PNGase F digest (Figure S6). Similar blots revealed the presence of the J‐chain (Figure 3b) and the single polypeptide VHH‐IgA and proteolysed version of it (Figure 3c). Quantifying the SC, J‐chain and VHH‐IgA signals after SDS‐PAGE in reducing conditions further revealed that there was no clear correlation between the sSIgA amounts as found by ELISA (Figure 2e,f) and the accumulation levels of either the SC, J‐chain or VHH‐IgA component (Figure 3a–c).

Further, we attempted to demonstrate the presence of assembled sSIgA complexes by SDS‐PAGE in non‐reducing conditions (Figure 3d–f). We postulate that the faint band at ~250 kDa corresponds to covalently linked sSIgA complexes of four VHH‐Fc polypeptides, one J‐chain and one SC. For most samples, the blots showed similar relative amounts of assembled sSIgA as found in the sSIgA‐specific ELISA (Figure 2e,f). Discrepancies found in the band intensities between ELISA and western blot could be attributed to the likely presence of functional, non‐covalently‐linked sSIgA, which is detectable by ELISA but disaggregated during the western blot procedure, and to the normalisation process applied to the OD values in the ELISA. Nevertheless, the high proportion of SC, J‐chain and VHH‐IgA found in other forms than in the sSIgA complexes is remarkable. For instance, apart from complexed with sSIgA, SC could be found as single polypeptide and as a postulated dimer in different glycoforms (Figure 3d). This was different for the J‐chain: in reducing conditions, the J‐chain was present at the expected molecular weight (Figure 3b), but in non‐reducing conditions, the J‐chains were detected in high‐molecular weight complexes, covalently linked among themselves or with antibody chains or with endogenous proteins, while no free J‐chain molecules were detectable (Figure 3e). Finally, the VHH‐IgA main bands corresponded to monomeric and single VHH‐IgA polypeptides and truncated or glycosylated versions of these molecules (Figure 3f). Differences in the level of glycosylation and proteolysis of the analysed molecules were observed between transformants, however, no specific patterns could be assigned to any specific group.

### Heritability of sSIgA production

**Figure 4:**
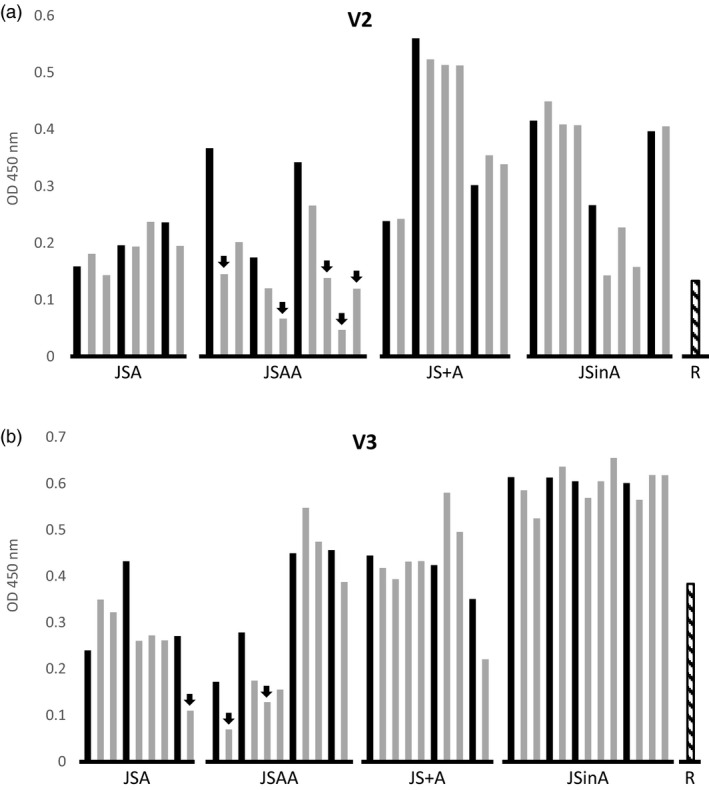
Heritability of sSIgA accumulation. For each strategy (JSA, JSAA, JS+A and JSinA), the OD (450 nm) values at a specific dilution in the sSIgA‐specific ELISA of seed extracts of three to four selected T2 parents and the derived homozygous offspring (one to four indicated with grey bars immediately at the right of the parent indicated in black) were obtained for the V2 (a) and V3 (b) VHH‐IgA variants. The reference (R) corresponds to the best sSIgA‐producing lines obtained by Virdi *et al*. (2013) (sV2A8 for V2 and sV3A40 for V3). The arrows indicate the T3 progenies that show a more than 50% reduction of sSIgA levels compared with the T2 parent.

To check the heritability of the expression, we compared the V2 and V3 sSIgA amounts in some segregating T2 seed stocks and their homozygous T3 progeny (Figure 4). Overall, the production of sSIgA in T2 and T3 seed stocks was comparable, except for several transformants in the JSAA series. In these T3 progeny seed stocks, both for the V2 and V3 series, the sSIgA amounts were decreased below 50% of the levels achieved by their parents. Thus, only when the three constructs were cloned in tandem in a single T‐DNA, they seemed more susceptible to gene silencing in subsequent generations, but we did not confirm this at the molecular level.

## Discussion

The transformation process to produce a hetero‐multimeric protein can follow different paths, leading to certain advantages and disadvantages related to workload, protein production level or transformation efficiency, among other parameters. To test this, we used sSIgA as model. Previously, Virdi *et al*. (2013) cloned the three encoding genes (J‐chain, SC and VHH‐IgA) in three different T‐DNA vectors, and transformants were obtained by triple cotransformation. Here, we evaluated four alternative strategies for the stable seed‐based production of sSIgA: a JSA single T‐DNA transformation, cotransformation of the JS and VHH‐IgA T‐DNAs, supertransformation of a selected VHH‐IgA transformant with the JS T‐DNA and crossing of selected JS and VHH‐IgA transformants. Table 2 summarizes the main advantages and disadvantages encountered for each strategy.

**Table 2:** Main advantages and disadvantages of each transformation strategy

	Single T‐DNA	Double cotransformation	Supertransformation	Parallel transformation & Crossing
Cloning process	Demanding	Easy	Easy	Easy
Transformation efficiency	High	Moderate	High	High
Plant manipulation steps	One	One	Two (or one†)	Two
Time to get T2 sSIgA‐accumulating plants	Moderate	Short	Long (or short†)	Long
High‐level sSIgA producers	Few	Medium	Many	Many
Selection markers	One	Two	Two	Two
Selection of single‐locus events	One locus	One or two loci	Two loci	Two loci
Selection of stable lines	Easy	Easy (if single locus)	Easy	Demanding

† Depending on whether a stable recombinant plant is available to be supertransformed.

In terms of complexity, the DNA assembly and cloning of the three components in a single T‐DNA vector turned out to be very difficult, because this T‐DNA vector is close to 20 kb, resulting in very low cloning efficiencies. Combining the genes for J‐chain and SC in one JS T‐DNA and the other VHH‐IgA component in another T‐DNA, made the cloning process much easier. Another important factor to consider when introducing multiple genes is to determine if a number of the genes can be reused to produce families of heteromeric proteins. For instance, in our sSIgA model, the J‐chain and SC remain constant within the same species, while only the VHH‐IgA differs for every different epitope (Janeway *et al*., 2001). Therefore, the reusability of the same J‐chain and SC to produce different SIgAs allows a simplification of the system by joining both expression cassettes (J‐chain and SC) in a single T‐DNA to use in a double cotransformation process (together with a VHH‐IgA bearing plasmid) or to get a stably transformed JS line producing the J‐chain and SC, which subsequently can be supertransformed with a VHH‐IgA bearing plasmid or crossed with a VHH‐IgA‐producing plant. Thus, plants with good expression of the constant elements can be reused in future projects with different VHH‐IgAs, whereas applying the single T‐DNA strategy implies that a new 20‐kb construct needs to be generated for each new VHH. Alternatively, the use of emerging cloning systems such as Golden Braid (Vazquez‐Vilar *et al*., 2017) or MoClo (Weber *et al*., 2011), both based on Golden Gate multiple assembly technology, may ease the assembly of huge constructs.

Another problem we encountered was the loss of the J‐chain expression cassette during clone establishment and propagation. These deletions are most likely the result of homologous recombination between the repetitive sequences flanking the different genes. Indeed, as a consequence of using identical transcriptional regulatory sequences, the three or four genes in the pMX‐JSA and pMX‐JSAA constructs, respectively, are flanked with stretches of more than 1400‐bp homology, and even in low‐recombination *E. coli* strains such as DH5α, these deletions were observed, as previously described (Bzymek and Lovett, 2001). This results in bacterial cultures with a mixed population of correct and truncated versions of the desired T‐DNA. Even though cultures were checked via restriction digestion before plant transformation, the occurrence of plants producing high concentrations of VHH‐IgA but no detectable levels of sSIgA suggests that these plants received a truncated version of the T‐DNA. These plants had to be discarded, resulting in a waste of time and resources. However, previous groups producing SIgA via the single T‐DNA method did not experience homologous recombination in their constructs (Ariaans *et al*., 2014; Juarez *et al*., 2013), and other groups, such as Ruiz‐Lopez *et al*. (2015) and Malik *et al*. (2015), managed to assemble up to six and four expression cassettes, respectively, without apparent complications. This suggests that the regulatory sequences used in this project favour the occurrence of recombination events. To lower the risk of homologous recombination, it is possible to work with a pool of different promotor and terminator sequences. DNA cloning systems such as Biobricks or GoldenBraid are specially designed for that purpose, and they allow to choose á *la carte* between a collection of promoters and terminators and combine them at will (Smolke, 2009; Vazquez‐Vilar *et al*., 2017). However, care has to be taken that these different transcriptional regulatory sequences are synchronised (switched on/off) in the same cells and at the same time.

Although cloning every gene in separate T‐DNA vectors is an approach that works well for transient expression (Juarez *et al*., 2013), for stable transformation, we do not advice to distribute the genes over more than two T‐DNAs because of the time and screening efforts required. For instance, the transformation efficiency registered for the triple cotransformation strategy by Virdi *et al*. (2013) (0.028%) was approximately 100‐fold lower as compared with the efficiencies achieved by introducing a single T‐DNA at a time (supertransformation and single T‐DNA strategies) and 20‐fold lower than that obtained by double cotransformation. Logically, the efficiency to obtain cotransformants after *A. tumefaciens*‐mediated transformation is inversely proportional to the number of different T‐DNAs that are required to integrate and varies depending on the plant species and target plant tissues (De Buck *et al*., 2000, 2009). The direct consequence of obtaining a lower number of cotransformants is the higher number of transformation projects that need to be started. In this project with Arabidopsis, a fourfold reduction in obtaining the desired transformants, when comparing the double cotransformation to the single T‐DNA strategies, is not an issue, but would be a serious limitation in plants recalcitrant to transformation (Birch, 1997). It should also be noted here that every T‐DNA commonly requires a different selection marker. However, a single T‐DNA needs only one selection marker, cotransformation and supertransformation require two selection markers.

Another point to consider is the time required to obtain sSIgA‐producing plants: whereas the single T‐DNA and cotransformation methods can give sSIgA‐producing plants in one plant transformation cycle, supertransformation and crossing require a first cycle to obtain stably transformed plants, and a second cycle for supertransformation or crossing. This extra cycle thus results in a substantial increase in time and efforts required to obtain the desired sSIgA‐recombinant plants. Taking into account that cloning a single T‐DNA with several constructs takes more time than cloning simple T‐DNA vectors, we conclude that cotransformation is the fastest and least work‐demanding option to obtain transformants producing sSIgA. On top of that, transforming plants such as tobacco and soybean may ease biomass upscaling of sSIgA‐transformed lines, but it comes at the cost of longer life cycles and more demanding tissue culture requirements to obtain the transformants. For instance, Ma *et al*. (1995) could stably produce up to 500 μg of SIgA per gram of fresh weight in the easy‐to‐scale tobacco leaves, however the process required (i.e. the sequential crossing of four transgenic tobacco plants) implied years of work until the first plant producing SIgA was obtained. On the other side, in plant species such as duckweed, in which a transformation process takes only a few weeks (Yamamoto *et al*., 2001), Ariaans *et al*. (2014) reached an SIgA production level of 16% of the total soluble leaf protein by transforming all the constructs in a single T‐DNA.

By determining the assembled sSIgA levels by ELISA and Western blot analysis, we were able to determine the variability in expression within each group and more importantly the number of high‐level sSIgA producers in each group. We found that the single T‐DNA transformants (JSA and JSAA) overall had the lowest performance in sSIgA accumulation, followed by cotransformation that only performed slightly better in one of the two VHH‐IgA variants. The low expression level of the three transgenes within one T‐DNA (JSA) might be due to the triple repeat of the regulatory elements, making them more sensitive to transcriptional gene silencing and position effects (Matzke and Matzke, 1998). Indeed, adding a second VHH‐IgA expression cassette to the single T‐DNA (JSAA) did not provide higher sSIgA levels. On the contrary, the transformants selected for the highest accumulation of sSIgA suffered a drastic reduction in the assembly levels of this molecule in the next generation most likely because of gene silencing effects. It has been described how the transgene copy number, the transgene expression level and the repetitive use of DNA sequences increase the occurrence of silencing (Assaad *et al*., 1993; Lessard *et al*., 2002; Matzke and Matzke, 1998). Moreover, the high frequency of silencing in the single T‐DNA transformants might be related to the floral dip transformation method for which it is known that multiple T‐DNA copies are frequently integrated (De Buck *et al*., 2009). Thus, using another transformation method or using a rapid screen for single‐copy T‐DNA inserts might improve the expression performance of transformants with T‐DNAs encoding all the components of a hetero‐multimeric protein. On the other hand, the results clearly demonstrate that supertransformation and parallel transformation followed by crossing are the best suited strategies to find excellent sSIgA producers among a limited number of transformants, most likely because of the independent selection of good events for both T‐DNAs. Indeed, if an optimal event occurs in about 1 of 20 transformants, then the probability of optimal expression from both T‐DNAs will only be found in 1 of 400 transformants. It is indeed known that transgene expression levels are variable but also, especially for single‐copy inserts, to a high degree heritable (De Buck *et al*., 2004). Therefore, after crossing two parentals with high‐expressing transgenes, hybrids with high sSIgA levels can be obtained, whereas, upon supertransformation, the first generation transformant is selected and screened for the best expression, and subsequently the best expressor for the second T‐DNA is selected among the second generation transformants, increasing the chance to isolate a transformant in which all transgenes have high expression rates. However, a more detailed look at the expression level of each component via western blot revealed that we are far from understanding the most suitable ratios for the assembly of sSIgA. The western blot results show how, even in the selected top sSIgA‐expressing seeds, several components are not covalently assembled in a functional sSIgA. VHH‐IgA and SC are also found as single polypeptides and as dimers in their glycosylated and non‐glycosylated form, truncated versions of VHH‐IgA are present as previously described by Virdi *et al*. (2013), and the J‐chain is covalently linked into aggregates that might limit the amount of free J‐chains available to form sSIgA. A bibliographic research showed us how the described effects have been experienced previously by other authors, such as the proteolysis of IgA (Nakanishi *et al*., 2017; Paul *et al*., 2014; Wieland *et al*., 2006), the simultaneous presence of different glycoforms (Juarez *et al*., 2013; Wieland *et al*., 2006) and the proteolysis of the SC (Ma *et al*., 1995; Nakanishi *et al*., 2017), the latter not observed here. These results emphasize the need to thoroughly study molecule stability and protein interactions when working with hetero‐multimeric proteins.

Another important aspect is the ease with which stable lines can be generated from the selected transformants by reaching homozygosity of the inserted T‐DNA(s). This is straightforward if there is only a single locus involved: then one in four of the segregating T2 seeds will be homozygous. A single‐locus insert is also frequently found after cotransformation, because cotransformed T‐DNAs frequently cointegrate at the same genetic locus (De Neve *et al*., 1997). On the other hand, supertransformation and stacking two events by crossing automatically involves two genetic loci. However, whereas screening for a double homozygous plant among the progeny of a double hybrid requires intensive screening, getting double homozygous plants in the supertransformation strategy is simple if a homozygous plant is used for supertransformation.

In conclusion, single T‐DNA and cotransformation approaches may represent the fastest options to obtain several transformants in a screening project. However, cotransformation gives less problems during DNA cloning, single T‐DNA cloning requires only one selection marker and single‐locus homozygous plants are easy to obtain. On the other hand, supertransformation and crossing are more time‐demanding but show an increased chance to obtain good sSIgA producers. When the aim is to generate a production platform for a library of sSIgAs, the best approach seems to be to first select a good stable, homozygous line producing high levels of the constant J‐chain and SC, and then to use this line to supertransform with several VHH‐IgAs to finally obtain a homozygous lines producing high levels of sSIgAs.

In all, our work here gives a clear overview of the advantages and disadvantages of transformation strategies that can be used for complex protein production in plants, and this can facilitate and guide important decisions about how to obtain high expression of hetero‐multimeric proteins in different plant production systems.

## Experimental procedures

### Construction of T‐DNAs for in‐seed sSIgA expression

All cloning experiments were performed in *E. coli* DH5α. The different transgenes required for in‐seed sSIgA production are described in Virdi *et al*. (2013). Briefly, the fusion VHH‐IgA and the porcine SC expression cassettes were introduced in the pPhasGW destination vector (Morandini *et al*., 2011), which has the *nptII* gene for kanamycin resistance, resulting in the expression vector pX‐A and pX‐S, while the porcine J‐chain expression cassette was contained in the pBm43GW,0 plasmid, which has a *bar* gene that confers resistance to phosphinothricin (PPT; Karimi *et al*., 2005), to obtain pX‐J. The combination of restriction enzymes *Xba*I, for pX‐S, and *Avr*II, for pX‐J, served to transfer the SC expression cassette from the original plasmid to pX‐J, to obtain pMX‐JS with both transgenes in tandem. Because *Xba*I and *Avr*II are isocaudomers, the resultant sequence lost the reassembled recognition sites. Then, the same combination of restriction enzymes was used to transfer the VHH‐IgA expression cassette for the V2 and V3 VHH‐IgA variants from the pX‐A to the pMX‐JS plasmid, to obtain the pMX‐JSA plasmid with all three components in tandem in a single T‐DNA. Unexpectedly, a plasmid with a double copy of the VHH‐IgA expression cassette (pMX‐JSAA) was also found.

### *Agrobacterium*‐mediated transformation via floral dip in *A. thaliana*

The T‐DNA plasmid vectors obtained were transformed into the *Agrobacterium* strain C58C1Rif^R^ with the helper plasmid pMP90 (Koncz and Schell, 1986) via electroporation (Shen and Forde, 1989). The *Agrobacterium* strains bearing the correct plasmids were used for floral dip of Col‐0 Arabidopsis plants, as described in Clough and Bent (1998). Transformants were selected on Murashige and Skoog agar medium supplemented with nystatin (50 mg/L), vancomycin (750 mg/L) and PPT (10 mg/L) and/or kanamycin (50 mg/L), depending on the selection marker of the plasmids used. Finally, single‐locus T2 transformants were identified and homozygous T3 seed stocks were obtained by segregation analysis as described previously (De Jaeger *et al*., 2002) for the heritability study.

### Characterization of J‐chain and SC transformants

The seed protein extraction was performed as described by De Jaeger *et al*. (2002). The levels of J‐chain and SC in the soluble extract were determined by SDS‐PAGE using 4%–15% Mini‐PROTEAN^®^ TGX™ Precast Protein Gels (Biorad, Hercules, CA, USA) under reducing conditions. Then the proteins were blotted onto a polyvinylidene difluoride membrane using the Trans‐Blot^®^ TurboTM Transfer System (BioRad). The SC was detected with mouse anti‐porcine SC (Thermo Fisher MA1‐80544, Waltham, MA, USA) followed by anti‐mouse IgG conjugated to horseradish peroxidase (HRP; GE Healthcare GENXA931). The porcine J‐chain was detected with mouse anti‐human J‐chain (Thermo Fisher MA1‐80527) followed by anti‐mouse IgG conjugated to HRP (GE Healthcare GENXA931, Chicago, IL, USA). Recombinant proteins were visualized and quantified using the Bio‐Rad ChemiDoc™ Imaging System (Bio‐Rad) and the Image Lab™ software (Biorad). A PNGase F (New England Biolabs, Ipswich, MA, USA) glycosidase treatment was performed in some samples to study the glycosylation state of SC, following the manufacturer's instructions.

### Crossing to obtain sSIgA‐producing hybrids

Four stable, high‐accumulating JS transformants were selected as mother plants to be crossed with the pollen of stably transformed lines producing the highest level of V2 and V3 VHH‐IgA as isolated by Dr. Virdi (Virdi *et al*., 2013). After the crossing process, the F1 hybrids were selected on Murashige and Skoog agar medium supplemented with PPT (10 mg/L) and kanamycin (50 mg/L), and were then propagated to harvest the segregating F2 seeds for subsequent protein analysis.

### Characterization of T2 and F2 sSIgA‐producing seeds

The levels of antigen‐binding antibodies in seed protein extracts from the T2 and F2 seed stocks were determined by ELISA with immobilized FaeGac (F4‐fimbriae most abundant adhesin). The wells were incubated with serial dilutions of 1 mg/mL seed protein extract (commonly 1/10, 1/40, 1/160 and 1/640) made in 2% skimmed milk in PBS. In the sSIgA‐specific ELISA setup, sSIgA complexes binding to FaeG were detected by using a mouse anti‐porcine SC (Thermo Fisher MA1‐80544) followed by an anti‐mouse IgG antibody conjugated to HRP (GE Healthcare GENXA931). In the VHH‐IgA‐specific ELISA setup, all different forms of IgA that bind to FaeG were detected by applying an anti‐pig IgA conjugated to HRP (Biorad AAI40P). TMB (3, 3′, 5, 5′ tetramethylbenzidine, Sigma) was used as substrate for the HRP, followed by adding 0.18 m H_2_SO_4_ 45 min post‐incubation to stop the reaction. Finally, both ELISAs were measured at 450 nm in a VERSAmax ELISA microplate reader (Molecular Devices). The results were normalized against a reference provided by Dr. Virdi (Virdi *et al*., 2013). After the analysis, the best sSIgA‐accumulating transformants were used in a titration experiment to calculate precisely the relative accumulation of sSIgA. In the assay, the sSIgA‐specific ELISA setup was applied to a broader serial dilution of the samples (1/80, 1/160, 1/320, 1/640, 1/1280, 1/2560, 1/5120 and 1/10240). End point titres were determined within the threshold of two times the average of a blank sample and then transformed into relative units compared to the dilution value recorded for the reference.

The protein seed extracts were also evaluated via western blots after SDS‐PAGE, similar to the evaluation of the JS transformants. The VHH‐IgA and the VHH‐IgA‐derived complexes were detected with an anti‐pig IgA antibody conjugated to HRP (Biorad AAI40P), while the same antibodies as used for the sSIgA‐specific ELISA were used for SC detection.

### Isolation of stable lines producing sSIgA

The best sSIgA expressors in T2 seed stocks among the JSA, JSAA, JS+A, and JSinA transformants for both V2‐ and V3‐containing VHH‐IgAs were propagated to the next (T3) generation to obtain stable lines. To simplify the procedure, a segregation analysis was applied and only single‐locus transformants (i.e. showing a 3:1 ratio of resistant to susceptible transformants) were kept. Analysis of the T3 offspring of these allowed to identify the homozygous lines. On the other hand, the F1 hybrids resulting from the crossing were propagated to F2 seed stocks, but stable lines based on screening for homozygosity of both loci (1/16) were not isolated.

## Author contributions

A.D. conceived the idea. J.P., V.V. and A.D. designed the research; J.P. performed research; J.P., V.V. and A.D. analysed the data; and all authors were involved in writing the manuscript.

## Conflict of interest

The authors declare no conflict of interest.

## Supporting information


**Figure S1** T‐DNA constructs for in‐seed sSIgA production.
**Figure S2** Homologous recombination event registered during the DNA cloning process.
**Figure S3** Screening of JS transformants by reducing western blots and immune‐detection of SC and J‐chain.
**Figure S4** ELISA setups.
**Figure S5** Relative ratios of sSIgA and VHH‐IgA amounts in seed extracts of transformants obtained by different transformation strategies.
**Figure S6** PNGase F analysis of SC after reducing PAGE.
