# Supplementary material for: Transformation strategies for stable expression of complex hetero‐multimeric proteins like secretory immunoglobulin A in plants
Source: Plant Biotechnol J. 2019 Mar 5;17(9):1760–9. doi: 10.1111/pbi.13098 (PMC6686127; doi:10.1111/pbi.13098)
Supplement: Supplementary file 1 — Figure S1 T‐DNA constructs for in‐seed sSIgA production. Figure S2 Homologous recombination event registered during the DNA cloning process. Figure S3 Screening of JS transformants by reducing western blots and immune‐detection of SC and J‐chain. Figure S4 ELISA setups. Figure S5 Relative ratios of sSIgA and VHH‐IgA amounts in seed extracts of transformants obtained by different transformation strategies. Figure S6 PNGase F analysis of SC after reducing PAGE. [file PBI-17-1760-s001.docx]

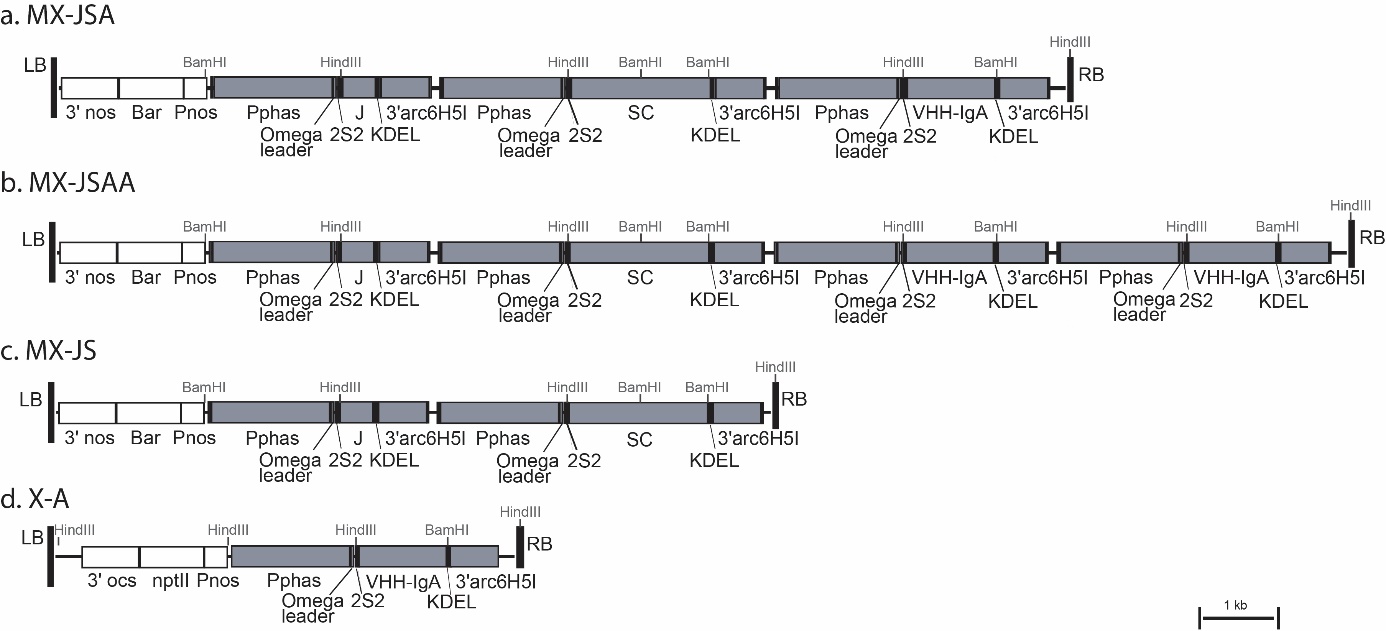


**Figure S1.** T-DNA constructs for in-seed sSIgA production. Representation (drawn to scale) of the single gene expression (X) and multiple gene expression (MX) T-DNA constructs for in-seed production of sSIgA. Each T-DNA construct (LB – left border, RB – right border) contains one to three different expression cassettes next to the selection marker. Transcription of the in-tandem oriented J-chain and secretory component (SC), both present in MX-JSA (a), MX-JSAA (b) and MX-JS (c), and VHH-IgA, present in X-A (d) , MX-JSA (a) and, two times, in MX-JSAA (b), is driven by the seed-specific Phaseolin promoter (Pphas), initiated with the 5’ tobacco mosaic virus untranslated region (Omega leader), and terminated by the 3’ arcelin terminator sequence (3’arc6H5I). At the translational level, the J-chain-, SC- and VHH-IgA-encoding sequences are fused with the signal peptide-encoding sequence of the 2S2 seed storage protein at the aminoterminal end, and with the endoplasmatic retention motif (KDEL) at the carboxyterminal end. The phosphinothricin herbicide resistance (Bar) gene (present in MX-JSA, MX-JSAA and MX-JS) and the kanamycin resistance (nptII) gene (present in X-A) are flanked both with the constitutive promoter of the nopaline synthase gene (Pnos), and the terminator of the nopaline synthase gene (3’nos) or the octopine synthase gene (3’ocs), respectively. BamHI, recognition site for BamHI restriction enzyme; HindIII, recognition site for HindIII restriction enzyme.

**
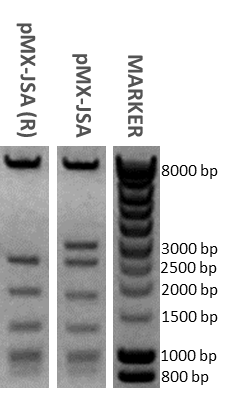
**

**Figure S2.** Homologous recombination event registered during the DNA cloning process. Example of a homologous recombination event (R) detected during the cloning of the pMX-JSA plasmid by applying the combination of restriction enzymes HindIII and BamHI. The absence of the band at ˜3000 bp indicates the absence of the J-chain fragment because of a homologous recombination event.


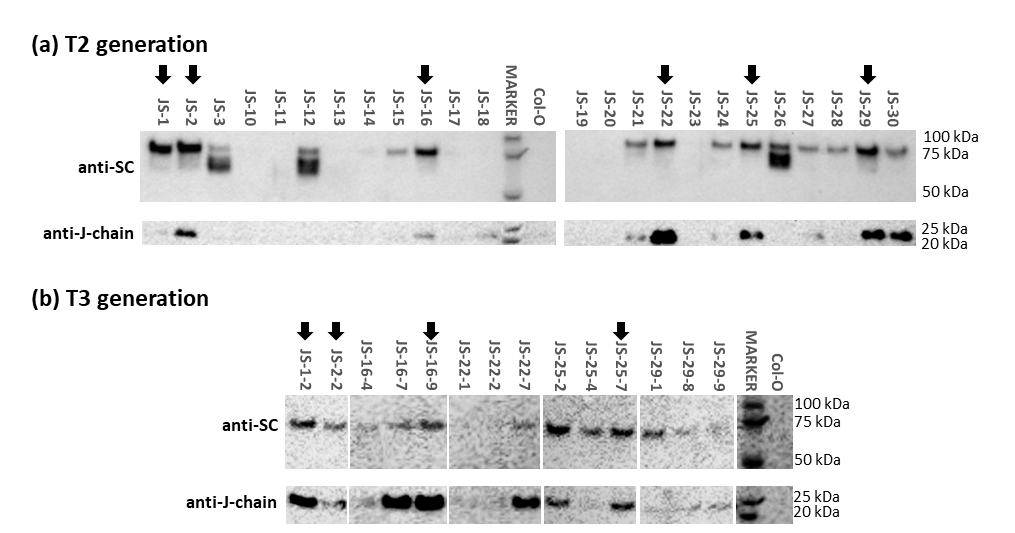


**Figure S3.** Screening of JS transformants by reducing western blots and immune detection of the SC and J-chain. (a) Analysis of 24 T2 JS transformants. The six transformants indicated with an arrow were propagated and screened for single-locus inserts. (b) Analysis of the single-locus, homozygous T3 offspring. The arrows indicate the transformants selected to perform the crossing.


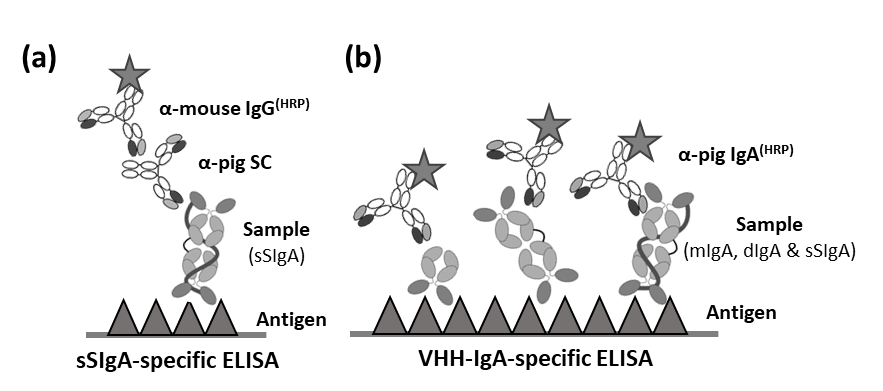


**Figure S4.** ELISA setups. (a) Setup used to measure the amount of fully assembled sSIgA. First, the well-coated FaeG-specific antigen is recognized by the V2-VHH of the sSIgA in the extract. Then, the SC of the assembled sSIgA is recognized by an anti-porcine SC, followed by an anti-mouse IgG conjugated to horseradish peroxidase (HRP*)*. (b) Setup used to detect all forms of VHH-IgA (mIgA, dIgA and sSIgA) in the extract. First, the well-coated FaeG-specific antigen is recognized by the V2-VHH in the VHH-IgA fusion and then, a polyclonal anti-porcine IgA antibody conjugated to HRP is used to detect the Fc part of the VHH-IgA.


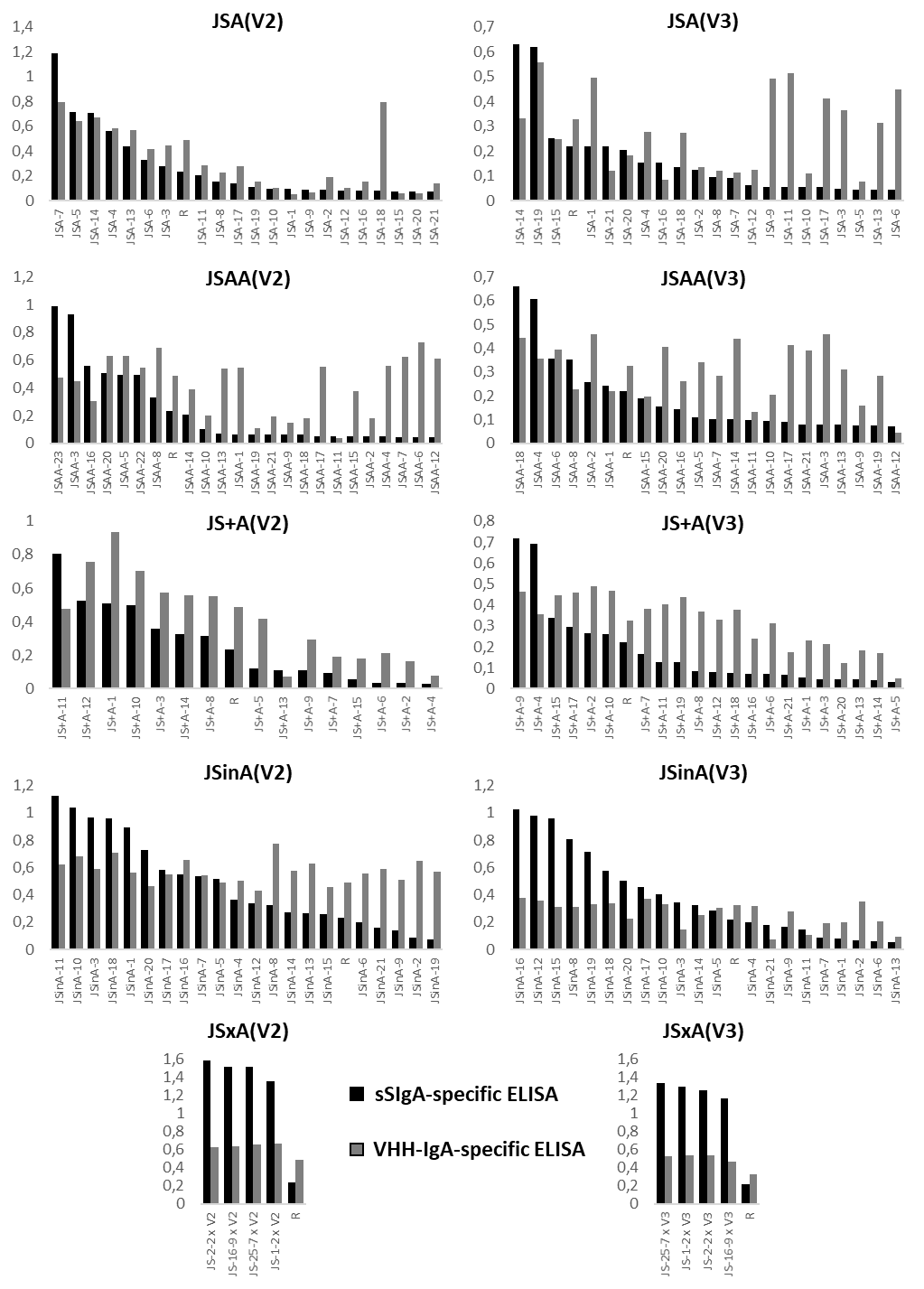


**Figure S5.** Relative ratios of sSIgA and VHH-IgA amounts in seed extracts of transformants obtained by different transformation strategies. The amounts were quantified by determining the OD values obtained for each transformant at a specific dilution in both sSIgA-specific (in black) and VHH-IgA-specific (in gray) ELISAs, which were then normalized using a reference (R) extract made from the sSIgA-producing lines, sV2A8 for the V2 and sV3A40 for the V3 variant described by Virdi *et al.* (2013). The samples have been grouped based on the transformation group and ordered according to the sSIgA accumulation levels.


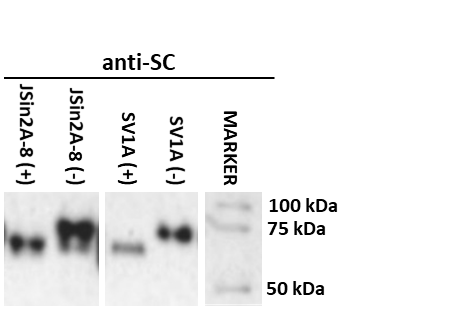


**Figure S6.** PNGase F analysis of the SC after reducing PAGE. Two samples of sSIgA-expressing transformants were compared before (-) and after (+) PNGase F treatment, followed by a western blot showing SC detection. The ~70‑kDa band in JSin2A-8 and in SV1A (corresponding to a V1 VHH-IgA reference sample (Virdi *et al*., 2013) before PNGase F treatment (-) is resolved to a SC band of ~65 kDa after PNGase F treatment (+).
